# Sleeping Beauties: Horizontal Transmission via Resting Spores of Species in the Entomophthoromycotina

**DOI:** 10.3390/insects9030102

**Published:** 2018-08-14

**Authors:** Ann E. Hajek, Donald C. Steinkraus, Louela A. Castrillo

**Affiliations:** 1 Department of Entomology, Cornell University, Ithaca, NY 14853, USA; 2 Department of Entomology, University of Arkansas, Fayetteville, AR 72701, USA; steinkr@uark.edu; 3 USDA ARS, Robert Holley Center for Agriculture & Health, Ithaca, NY 14853, USA; Louela.Castrillo@ars.usda.gov

**Keywords:** Entomophthoromycotina, insect pathogens, zygospore, azygospore, pre-death behavior change, field persistence, epizootics

## Abstract

Many of the almost 300 species of arthropod-pathogenic fungi in the Entomophthoromycotina (Zoopagomycota) are known for being quite host-specific and are able to cause epizootics. Most species produce two main types of spores, conidia and resting spores. Here, we present a review of the epizootiology of species of Entomophthoromycotina, focusing on their resting spores, and how this stage leads to horizontal transmission and persistence. Cadavers in which resting spores are produced can often be found in different locations than cadavers of the same host producing conidia. Resting spores generally are dormant directly after production and require specific conditions for germination. Fungal reproduction resulting from infections initiated by *Entomophaga maimaiga* resting spores can differ from reproduction resulting from conidial infections, although we do not know how commonly this occurs. Reservoirs of resting spores can germinate for variable lengths of time, including up to several months, providing primary infections to initiate secondary cycling based on conidial infections, and not all resting spores germinate every year. Molecular methods have been developed to improve environmental quantification of resting spores, which can exist at high titers after epizootics. Ecological studies of biological communities have demonstrated that this source of these spores providing primary inoculum in the environment can decrease not only because of germination, but also because of the activity of mycopathogens.

## 1. Introduction

**Figure 1:**
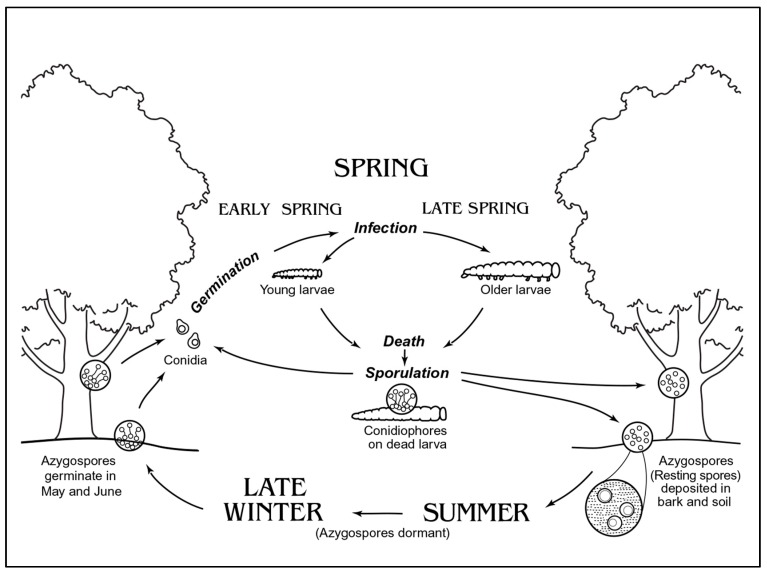
Life cycle of entomophthoralean fungus *Entomophaga maimaiga* infecting gypsy moth, *Lymantria dispar*. Generally, conidia are formed from infected younger larvae after death and resting spores are formed in the late spring/early summer within later instar larvae that often die on tree trunks. Resting spores within cadavers of later instars enter dormancy when drying. Cadavers containing resting spores eventually decompose on the soil surface and most resting spores persist in the surface layers of the soil during summer and winter, when the non-susceptible gypsy moth adults and eggs are present. In spring, when gypsy moth eggs begin to hatch, resting spores begin germinating to cause primary infections, leading to cycles of secondary infections that eventually result in production of resting spores that are dormant. Resting spores germinate over several months and not all resting spores in the soil germinate every year, so a reservoir of resting spores persists in the soil. (Graphic by Frances Fawcett).

Diverse pathogens persist in the environment as long-lived forms in reservoirs, with perhaps the best documented example being the bacteria that causes anthrax (*Bacillus anthracis*) [1]. Long-lived forms allow persistence in the environment over periods when host stages that are present are not susceptible or prevailing environmental conditions are not ideal for successful pathogen transmission and development. It has been hypothesized that having such means for persistence allows these pathogen species to retain high virulence, even allowing all hosts in that location to be killed [2,3,4]. Of course, horizontal transmission is required for these persistent stages to infect hosts. Horizontal transmission, however, is more risky than vertical transmission and becoming active and successfully infecting hosts during the correct periods of time are critically important to pathogen survival and continued persistence. Most species in the fungal subphylum Entomophthoromycotina produce long-lived spores that persist in the environment to initiate infections in insects when susceptible host stages are present (Figure 1).

**Figure 2:**
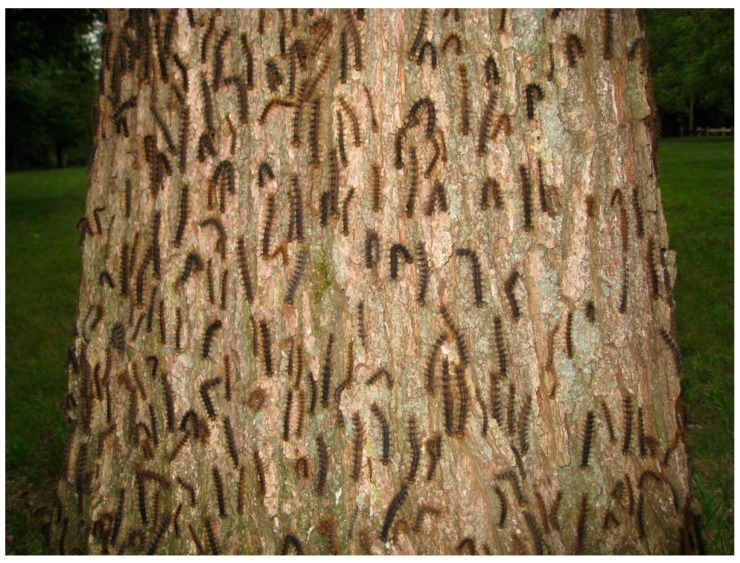
Epizootic in a gypsy moth, *Lymantria dispar*, population, principally caused by *Entomophaga maimaiga*. The majority of these cadavers are filled with *E. maimaiga* resting spores (azygospores), although some could have died from *Lymantria dispar* multiple nucleopolyhedrovirus or co-infections of these two pathogens (photo by Heather Faubert).

**Figure 3:**
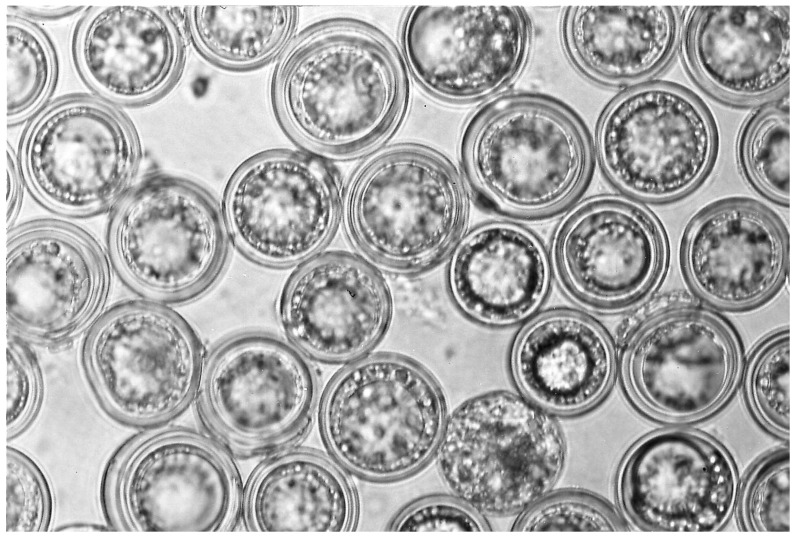
Resting spores (azygospores) of *E. maimaiga* that were formed within later instar gypsy moth larvae (*Lymantria dispar*) killed by this fungus, and which fill the cadaver (~30–32 μm diameter). The thick walls and regular shape of dormant resting spores are visually attractive (i.e., sleeping beauties); this form of resting spore is not atypical among fungi in the Entomophthoromycotina.

The subphylum Entomophthoromycotina (Zoopagomycota) includes highly virulent species that are capable of causing epizootics (Figure 2) and have a specialized form for persistence. Among the >300 species of Entomophthoromycotina, most are obligate pathogens of arthropods with complex life cycles associated with their hosts. Most species in the Entomophthoromycotina produce at least two types of spores: conidia and either zygospores or azygospores (Figure 3) or a mixture of these latter. While conidia are generally immediately able to germinate and infect hosts, azygospores or zygospores (commonly referred to as resting spores; Figure 3) are thought to enter dormancy after production, becoming environmentally persistent forms. It is not uncommon that resting spores are not included in species descriptions, as only conidia have been found by the describers (e.g., [5]). However, we will explain why it is possible or probable that most species in Entomophthoromycotina produce both types of spores.

This is a review focusing on ecological aspects of resting spores made by species in the Entomophthoromycotina, with emphasis on horizontal transmission. We will not review all literature, but will especially explain research conducted since the review of entomophthoralean biology and ecology by Pell et al. [6], and the review of persistent forms of some Entomophthorales by Nielsen et al. [7].

## 2. Systematics and Habits

The systematics of the group Entomophthoromycotina has undergone major revisions, as new data from multigene analyses clarify phylogenetic relationships among genera traditionally identified in this group and with other basal fungi [8,9,10,11,12,13,14]. Traditionally, this group was placed in the phylum Zygomycota, based on the shared characteristic of zygospore formation during the sexual cycle, after fusion of specialized hyphae called gametangia (but see below). Like most members of the Zygomycota, entomophthoralean fungi also have coenocytic hyphae (lacking cross walls or septa), in contrast to Ascomycota and Basidiomycota with septate mycelia [10]. Analysis of the rRNA, *rpb1* and *tef1* by James et al. [9], however, showed that the group Zygomycota was polyphyletic (i.e., with shared characteristics, but derived from two or more ancestral forms not common to all members). In a comprehensive review of the kingdom Fungi by Hibbett et al. [11] in 2007, based on available molecular phylogenetic analyses, a proposal was made to distribute taxa traditionally placed in Zygomycota among four subphyla *incertae sedis* (i.e., no phylum has been assigned, or the broader relationship is unknown), including the new subphylum Entomophthoromycotina for fungi in the order Entomophthorales.

In 2012, Humber [8] proposed the creation of a new phylum, Entomophthoromycota, and discontinued use of Entomophthoromycotina, based on data from additional phylogenetic studies utilizing more loci and sampling from more representative taxa of entomophthoralean fungi that showed this group was monophyletic (i.e., with shared derived characters; descended from a common ancestor) [12,13]. Using a combination of molecular data and traditional taxonomic characters (i.e., nuclear characters, details of their mitoses, and morphology of primary and secondary conidia), a new classification was proposed, with the subphylum Entomophthoromycotina containing three classes, namely, Basidiobolomycetes, Neozygomycetes, and Entomophthoromycetes, and with each class consisting of only one order ([8]; see Table 1). The first two classes consisted of a single family each, Basiodiobolaceae and Neozygitaceae, respectively. The Entomophthoromycetes had four families, three of which contained arthropod pathogenic species (Table 1). Also included in this proposal was the placement of the genera *Eryniopsis* and *Tarichium* as *incertae sedis*. *Eryniopsis* species identification had been based on morphological characters only, while *Tarichium* was a form genus, based on the absence of conidia. Further, multi-gene sequence data separated species of the former between two subfamilies, Entomophthoroideae and Erynioideae, in Entomophthoraceae [8,13]. Species in both genera need to be analyzed further by single- and multi-locus phylogenetic reconstructions to determine possible synonyms or placement of taxa in the correct genus (e.g., [15]).

A more recent study by Spatafora et al. [14], utilizing genome-scale data to analyze zygomycete fungi, proposed the creation of a new phylum, Zoopagomycota, with subphyla Entomophthoromycotina, Kickxellomycotina, and Zoopagomycotina. The authors rejected use of the name Zygomycota for this phylum, because zygospore formation is not a synapomorphy, but a sympleisiomorphic trait (i.e., trait shared by common ancestors of Zoopagomycota and other phyla). The phylum name Zoopagomycota was selected over other possible names, including Entomophthoromycota, because of its broader and more inclusive meaning [14]. This new proposal retained the three classes in the subphylum Entomophthoromycotina proposed by Humber [8].

Studies on the molecular phylogeny of Entomophthoromycotina by Gryganski et al. [12,13], using multi-gene sequences (rRNA, *rpb2* and mtssu) from 63 species representing 14 genera, showed that lineages inferred from molecular data were in agreement with groupings based on traditional taxonomic characters used to identify these fungi. These characters included morphological and ultrastructural features: type of conidiophores, type of primary conidia, type of secondary conidia, type of resting spores, and type of nuclear division [8,12]. Based on morphology, characteristics of the primary and secondary conidia were suggested as generally more useful than those of resting spores for species identification. However, resting spore characters such as shape, size, color, ornamentation, wall structure, and mode of germination are also key features in species identification in some genera (and see Section 3.2) [16,17]. For more information on traditional taxonomic characters and for identification keys to families, genera and species of Entomophthoromycotina, in particular arthropod pathogens in the order Entomophthorales, see Keller [18,19] and Keller and Petrini [20]. 

Most members of the Entomophthoromycotina are arthropod pathogens, with some plant pathogens, commensals, or soil saprobes. Among arthropod pathogens, another key character is the host, as many are host-specific, with only one known host or with a relatively narrow host range (i.e., hosts in the same family). The signs and symptoms of infection can also be quite useful in identifying the fungus or the etiological agent of disease. For example, flies in the families Anthomyiidae and Fanniidae infected with *Strongwellsea* spp. exhibit pathognomonic signs and symptoms (i.e., presence of a gaping hole in the ventral abdominal pleuron of adults, through which spores are dispersed from an internal hymenium, associated with the inability of infected female hosts to lay eggs [21]). Among the three classes of Entomophthoromycotina, most of the genera with insect pathogenic species are found in the class Entomophthorales, specifically in the families Ancylistaceae, Entomophthoraceae, and Meristacraceae (Table 1). Identification of these fungi requires knowledge of host species in addition to descriptions of diagnostic structures that vary at different stages of infection [17]. Thus, morphology, in addition to molecular data, provides robust and stable classifications. Additional phylogenetic studies, however, are still needed to resolve several issues in the systematics of Entomophthoromycotina, including revising the *Erynia*/*Furia*/*Pandora* complex and sorting the identification of species in the genera *Eryniopsis*, *Neozygites*, and *Tarichium* [13].

In the past, morphological structures associated with conidiation were the sole means for species identification. However, cadavers of hosts killed by entomophthoralean pathogens can sometimes contain only resting spores and, in these situations, the pathogen could not be identified based on conidial morphology. In such cases, the species were included in the ‘form genus’ *Tarichium*, which is a temporary arrangement. At present, there are 40 species in the form genus *Tarichium* [15]. In recent years, however, the first species that was only known from the resting spores was described. Molecular evidence was used to place this species, *Zoophthora independentia*, in the correct genus [15]. In this case, resting spores are formed after death of adults of a native North American crane fly species, and cadavers are subsequently attached to the undersides of leaves by pseudorhizomorphs. The conidial stages have never been found and it is assumed that either adult hosts producing conidia are dying somewhere that collectors have not yet found them or conidial stages are produced from larvae or pupae in the leaf litter and soil and have not yet been found. At this time, questions remain for species in the genus *Tarichium*: are there actually no conidial stages, or they are limited, or have researchers not been looking at the correct times and/or locations to find them?

## 3. General Biology

Fungi in the Entomophthoromycotina are described as having primary conidia that usually are formed externally on cadavers. Primary conidia may be uni- to multinucleate and are frequently forcibly discharged. Many species also form one or more types of secondary conidia (formed from the primary conidia), which may be forcibly discharged or passively dispersed. Secondary conidia can resemble primaries, although smaller, or can differ completely in appearance. One type of secondary conidia, called capilliconidia, are formed at the top of a thin stalk produced from the primary. Capilliconidia are sticky and adhere and subsequently infect when contacted by passing hosts. Resporulation can also proceed to tertiary conidia, but infectivity of this stage has not been proven. Primary and/or secondary conidia are infective, but this characteristic can vary by fungal species. Most species are also thought to form resting spores, either zygospores (after gametangial conjugation) or azygospores (no gametangial conjugation), with thick walls [8]. Germination studies suggest that resting spores themselves are not infective, but they germinate to produce infective germ conidia. Germ conidia can resemble primary conidia, but at least some studies have demonstrated differential reproduction after host infection by germ conidia [42]. Whether germ conidia should be considered a third type of spore for Entomophthoromycotina or a secondary spore produced by resting spores has not been discussed, as germ conidia are poorly known for most species.

**Figure 4:**
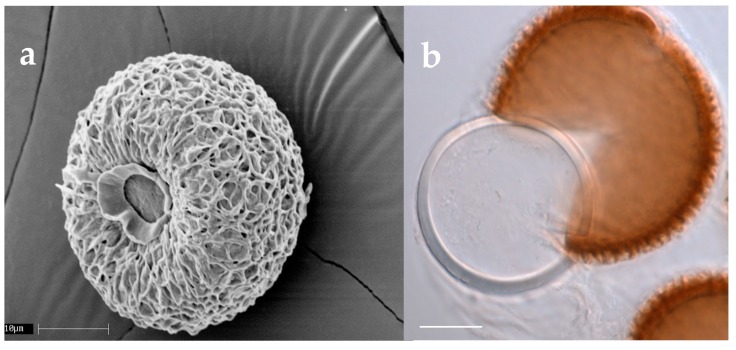
Resting spores of *Zoophthora independentia* (~44.5 μm diameter) [15]. (**a**) Scanning electron micrograph illustrating the surface of the opaque epispore. (**b**) Light microscope photo illustrating an epispore detached from the smooth, hyaline endospore within. Scale bars are 10 μm.

For many species, resting spores are produced within the body of the dead host, although for at least *Pandora dipterigena*, resting spores are produced externally on cadavers [5] and in *Neozygites floridana*, resting spores are produced within host mites when these hosts are still alive [43,44]. In the case of *E. maimaiga*, after host death the hyphal bodies round up on one end as they begin to become resting spores. Once round, the interiors of resting spores are granular and walls are thin, but this changes over the next 1–2 days while resting spores undergo maturation, until most resting spores have thick walls and contain large oil droplets [45,46]. There can be extreme diversity across species in the appearance of resting spores, but this has never been addressed as a taxonomic character, probably in part because resting spores are not known for many species. Among resting spores, some have no epispore while others have clear epispores that are ornamented or not, or some species have thick, opaque epispores that can be ornamented and colored. At least for the tipulid pathogen *Z. independentia*, the opaque epispore can be cracked and removed using pressure on a cover slip and the resting spore within has a thick wall and an appearance similar to some resting spores without epispores (Figure 4; [15]). While resting spores of most species are spherical, those in the Neozygitaceae can be ovoid and dark brown to black [20].

### 3.1. Resting Spores Versus Conidia and Species Identity

Most entomophthoralean species can produce two types of spores (conidia and resting spores) from one host individual. Sometimes, both conidia and resting spores are produced from one cadaver (e.g., *Entomophaga maimaiga*), but more commonly, only one type of spore is produced from the cadaver of one host individual. Usually, resting spores are formed under different conditions or host states than conidia (see Section 3.2).

In many ways, entomophthoralean pathogens that produce conidia and resting spores can resemble two different species. The locations of the host cadavers, the manipulations of the host behavior before and after death, the fungal structures such as rhizoid holdfasts, as well as the fungal reproductive forms differ so much that if only one form (i.e., type of spore) is found, this may be described as a separate species from the other form (see Section 3.3). When resting spores are formed within host stages that are hidden and not easily found (e.g., within the soil), resting spores might never have been found and have not been described for that species. Conversely, sometimes resting spore-bearing hosts are found but individuals of the same host species in which conidia are produced are not found. In such a case, the species has been placed in the genus *Tarichium*. However, it is possible that the conidial stages have already been described as a different species. This sets up a situation similar to Ascomycota, where one species might have two different names, which has occurred when ascomycete anamorph and teleomorph forms are disconnected. 

**Figure 5:**
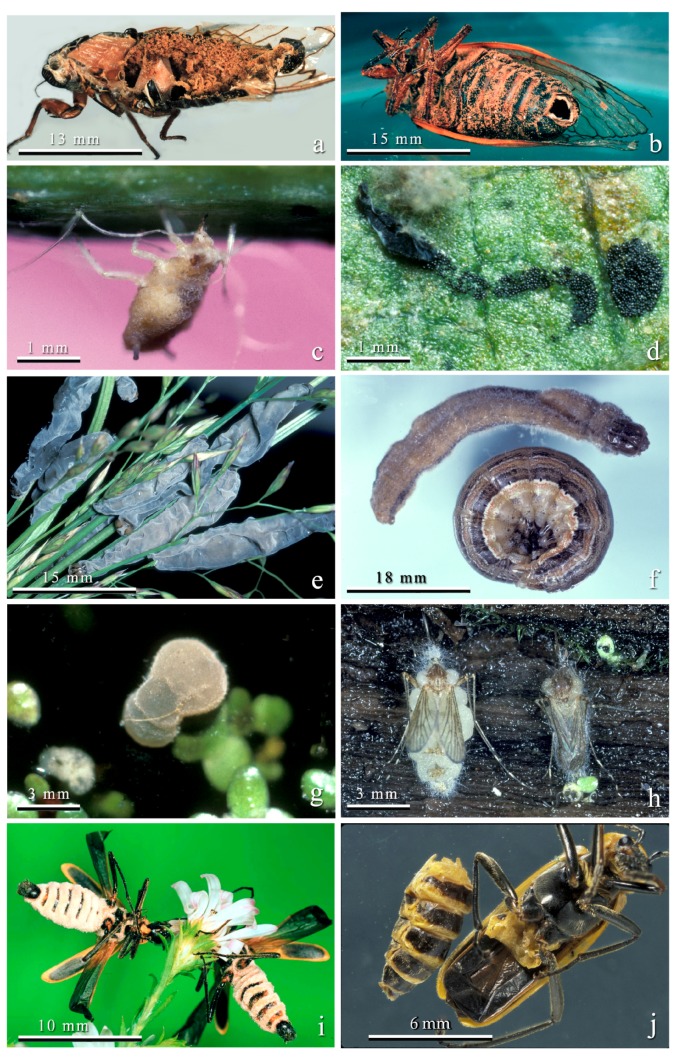
Insect-pathogenic entomophthoralean fungi. (**a**) Male *Magicicada septendecim* showing abdomen full of resting spores of *Massospora cicadina.* (**b**) *M. septendecim* showing abdominal hole through which *M. cicadina* resting spores are scattered as the living infected cicada flies. (**c**) Cotton aphid, *Aphis gossypii*, infected with the conidial stage of *Neozygites fresenii* held onto the leaf by mouthpart stylets. Primary conidia are explosively discharged into the air, onto the leaf, and onto adjacent aphids. (**d**) In contrast, when an infected *A. gossypii* produces the resting spore stage of *N. fresenii*, its body becomes fragile, liberating ca. 1000 dark black oval resting spores onto a leaf. Resting spores are not explosively discharged and enter the soil via rain or leaf fall. (**e**) True armyworms, *Mythimna unipuncta*, infected with the conidial stage of *Furia virescens*. Infected larvae climb up a grass stem, grip it with their prolegs, die, and discharge conidia. (**f**) In contrast, cadavers of *M. unipuncta* producing *F. virescens* resting spores die on the ground attached to the soil with numerous rhizoids. The top larva died and contained resting spores; the lower larva is an uninfected armyworm. (**g**) *Aedes fitchii* pupa infected with *Erynia aquatica* that produced conidia. Infected larvae and pupae that produce conidia float on the water surface and discharge conidia onto emerging adult mosquitoes. (**h**) In contrast, resting spores of *E. aquatica* were formed only in adult *Aedes* spp. Both of these adult cadavers producing resting spores were firmly attached by rhizoids to damp wood adjacent to snowpools. (**i**) *Chauliognathus pensylvanicus* cadavers producing the conidial stage of *Eryniopsis lampyridarum*. These beetles died attached to flowers by their mandibles. (**j**) In contrast, infected *C. pensylvanicus* producing resting spores were found on the ground with no outward signs of infection and their abdomens were filled with resting spores.

The potential disjunct occurrence of different types of spores demonstrates the importance of detailed studies over time of a host/pathogen in order to understand all the stages. For instance, in the case of *Erynia aquatica*, the first researchers to study this pathogen of *Aedes* spp. snowpool mosquitoes found only conidial stages (Figure 5g). They correctly hypothesized that resting spores must exist because the hosts were univoltine [35,47]. Later, this hypothesis was proven to be true when Steinkraus and Kramer [48] found and described the resting spores (Figure 5h).

### 3.2. Resting Spore Production

Empirical studies have repeatedly reported that resting spores are formed towards the end of a season, when few of the hosts will be present. Studies have demonstrated that numerous biotic and abiotic factors are associated with resting spore formation, but these can differ for different host/pathogen systems (Table 2). For example, *E. maimaiga* shifted from making conidia to making resting spores at warmer temperatures, as later larval instars of its univoltine host pupate in summer. In contrast, *Entomophthora muscae* and *Neozygites floridana* produced resting spores at colder temperatures, when hosts would no longer be active. In the case of *Eryniopsis lampyridarum* in soldier beetles, resting spores are produced when the adult hosts are abundant. 

### 3.3. Differential Locations of Cadavers Producing Resting Spores versus Conidia: Six Case Histories

Because the conidial stage of entomophthoralean fungi functions in short term dissemination of the pathogen to new hosts and conidia are not durable structures that survive many months or years, hosts producing the conidial stage must be held in situations where hosts are active. For example, conidia-producing cadavers of rhagionids infected by *Erynia ithacensis* are underneath leaves [58]; *Entomophthora scatophagae* producing conidia from dead yellow dung flies (*Scatophaga stercoraria*) are found on flowering grasses where other dung flies are perching [59]; or, in the case of *N. fresenii*, infected conidial stage aphids are attached by their mouthpart stylets to the plant (Figure 4c), and spores are explosively discharged onto adjacent aphids in the colony, onto leaves, or into the air [60].

Many arthropod pathogenic Entomophthoromycotina are known only from their conidial stages [61]. There are two reasons for this. First, hosts killed by the conidial stage often occur in highly visible situations, such as on plants (Figure 5e), held by rhizoids, legs, or mouthparts (Figure 5i). From elevated positions conidia can be discharged onto potential hosts. In contrast, hosts producing resting spores are not as conspicuous. Second, many studies on arthropod hosts of Entomophthoromycotina have not been carried out in detail over a long enough period to detect hosts with resting spores that may occur in older hosts or towards the end of the season.

With any entomophthoralean fungus that infects univoltine hosts, or multivoltine hosts that have an extended period when hosts are not available (such as during winter), there must be resting spores produced (or some other survival structure, e.g., *N. floridana* hyphal bodies within hibernating mites [44] or thick-walled conidia of the aphid pathogen *Pandora neoaphidis* [7]). Only careful studies over time with many specimens will result in finding the resting spore stage, as well as the conidial stage (or vice versa). Therefore, most species of Entomophthoromycotina must have a resting spore stage, but many have not yet have been discovered.

In order to find hosts containing resting spores, scientists must examine host populations over time, often focusing on older hosts. If the prevalence of hosts with resting spores is low, one must collect many hosts, perhaps hundreds both alive and dead, keep them and examine them for resting spores that may develop over time (e.g., [43,44]). Careful, thorough searches should also be made of the soil surface or other areas near where hosts dying of conidial stages are found, where host eggs are oviposited, or where the next generation of hosts will be found. Following these suggestions could result in fruitful discovery of resting spore stages, greater understanding of entomophthoralean ecology and possibly utilization of these pathogens in biological control or integrated pest management.

In this section, we will compare and contrast the conidial and resting spore stages of the following six species of fungi and their hosts:

*Massospora cicadina* and Periodical Cicadas (*Magicicada* spp.)
*Neozygites fresenii* and Cotton Aphids (*Aphis gossypii*)
*Furia virescens* and the True Armyworm (*Mythimna unipuncta*)
*Erynia aquatica* and *Aedes* spp. Mosquitoes
*Eryniopsis lampyridarum* and Soldier Beetles (*Chauliognathus* spp.)
*Entomophaga maimaiga* and Gypsy Moth (*Lymantria dispar*)

#### 3.3.1. *Massospora cicadina* Infections in Periodical Cicadas

This pathogen infects periodical cicadas in North America. Periodical cicadas (*Magicicada* spp.) have the longest known life cycles of any insects [62]. These cicadas emerge in broods that occur either every 13 years (in the south) or 17 years (in the north). Therefore, it was assumed that the resting spores of *M. cicadina* must remain dormant in the soil either 13 or 17 years in order to infect new broods of cicadas [63] (but see Section 4.3.3). Both the conidial stage and the resting spore stage are produced in adult cicadas. *Massospora cicadina* is one of the most interesting of the fungal pathogens because it turns its living hosts into dispersers of both conidia and resting spores. The fungus forms its spores within the abdomen of the living cicadas but the insects remain able to fly and behave relatively normally and spread conidia to new victims or resting spores to the soil for the long wait for the next brood [64].

After spending either 13 or 17 years in the soil sucking on roots of plants, several weeks before the mass emergence cicada nymphs burrow near the surface of the soil. It is during this time and during the actual emergence of nymphs from the soil that they probably encounter infective spores produced by germinating resting spores and become infected. Research is needed to determine exactly when and where cicada nymphs become infected when resting spores germinate. Nymphs infected via germinating resting spores result in adult cicadas producing the conidial stage [65]. Infected cicadas with the conidial stage interact with and infect new hosts. Cicadas infected by conidia produce the resting spore stage. As in the conidial stage, the infected cicadas that produce resting spores remain alive, lose part of their terminal abdominal segments but in this case the abdomen becomes filled with a mass of loose dry resting spores. As the host flies about the emergence area, resting spores are scattered randomly onto the soil (Figure 5a,b).

Cooley et al. [64] demonstrated that infected male periodical cicadas producing the conidial stage of the fungus exhibited “wing-flick” mating behaviors normally made only by female cicadas. This resulted in interactions where uninfected males attempted to mate with infected males, exposing more healthy males to infection. Somehow the fungal infection altered normal male behavior, resulting in increased spread of the pathogen. In contrast, male cicadas in which the fungus produced the resting spore stage did not exhibit this altered behavior.

#### 3.3.2. *Neozygites fresenii* Infections in *Aphis* spp.

*Neozygites fresenii* is an important pathogen of cotton aphids, *Aphis gossypii*, and other aphids in the USA and elsewhere [24]. It causes geographically and numerically vast epizootics in outbreak cotton aphid populations. Most infected individuals (both apterae and alatae) produce the conidial stage. Aphids producing conidia are held onto leaf surfaces by their mouthpart stylets alone (Figure 5c). The infection process is very complicated. Primary conidia are discharged from aphids, land on leaf surfaces, directly on aphids, or enter the air [66,67]. These aerial conidia can reach extremely high densities during epizootics and spread the pathogen widely and efficiently [66]. Primary conidia germinate to form capilliconidia on long slender stalks. Capilliconida are the infective stage and have a sticky apex called a mucoid hapteron that attaches them to new hosts.

In contrast, when *N. fresenii* forms resting spores the cotton aphid cadaver is filled with black, ovoid resting spores and the body of the aphid liquefies, leaving a film of resting spores on the leaf surface (Figure 5d). Rain and leaf fall result in resting spores becoming deposited on the soil. Resting spores survive the winter and the next season germinate and infect new aphid populations.

Ben-Ze’ev et al. [68] found that *N. fresenii* was an important pathogen of the citrus aphid, *Aphis citricola*, in Israel. They found that resting spores formed in aphids during the fall then germinated in the spring after a maturation/vernalization. Bitton et al. [69] found that *N. fresenii* overwintered as resting spores on the bark of citrus trees in Israel. These resting spores germinated in the spring in synchrony with the population increase of its host, *Aphis spiraecola*.

#### 3.3.3. *Furia virescens* in the True Armyworm

The true armyworm (*Mythimna unipuncta*) is a noctuid moth that is a serious pest of wheat, fescue, and other grasses [70]. It has many natural enemies including the fungus, *Furia virescens*. *Furia virescens* is a perfect example of how hosts producing the conidial stage of many Entomophthoromycotina are very obvious (Figure 5e), whereas the resting spore stages require greater effort to discover (Figure 5f). Infected larvae that produced the conidial stage died attached by their legs and prolegs to fescue plant stems a mean of 67 cm above the soil surface [70]. No rhizoids were formed and infected larvae were light grey and easily observed. Experiments demonstrated that larvae infected by conidia produced the resting spore stage [70]. Larvae with the resting spore stage were only discovered in the field by collecting live larvae beneath fescue plants and holding them for mortality. When *M. unipuncta* larvae collected from fescue were held in the lab, 21.6% produced resting spores when dying from *F. virescens* infections. The rest of the larvae either pupated or were killed by parasitoid wasps or unknown causes. Unlike the conidial stage, larvae producing resting spores died on the soil surface and large numbers of rhizoids attached the cadavers to the soil surface (Figure 5f). Without a diligent search of the soil surface or collection and rearing of large numbers of living larvae it would be impossible to find the cadavers containing the resting spore stage.

Because resting spores are the source of inoculum for epizootics in subsequent years, it is clear that resting spore stages are a valuable resource in biological control. It is unknown how agronomic practices in wheat and fescue fields could affect survival of resting spores of *F. virescens* or similar management in cotton fields could affect survival of resting spores of *N. fresenii*.

#### 3.3.4. *Erynia aquatica* Infections in *Aedes* spp. Mosquitoes

*Erynia aquatica* is the only member of the Entomophthoromycotina known to infect immature stages of Culicidae [35]. This pathogen occurs in *Aedes* spp. that breed in snowpools, or ephemeral pools, and thus it must have a way of surviving periods when no hosts are available [35]. Mosquito larvae are infected in the spring by germ conidia produced from resting spores formed the previous year in adult mosquitoes. Infected immatures die either as larvae or pupae and are found floating on the water surface discharging conidia (Figure 5g). These conidia infect adult mosquitoes. Infected adults die attached by rhizoids to damp logs in or near the water pools (Figure 5h). Adult mosquitoes that produce the long-lived resting spores after death can disseminate the fungus to new mosquito breeding sites if they disperse before dying [48].

There are several striking differences between the conidial and resting spore stages of *E. aquatica*. First, host stage: conidia were produced only from late larval and pupal stages, while resting spores were produced only from adults. Second, rhizoids were not produced from conidial-stage mosquito immatures, whereas copious rhizoids were formed from adults producing resting spores. The rhizoids held the adult hosts firmly attached to moist wood in the snowpools, such as sticks and logs. Third, infected immatures were found floating on the surfaces of snowpools while infected adults with resting spores were found attached to moist logs associated with the snow pools.

#### 3.3.5. *Eryniopsis lampyridarum* Infections in Soldier Beetles

One of the most interesting of insect pathogens is *Eryniopsis lampyridarum*. It appears to have a host range restricted to two species of Coleoptera in the Cantharidae: *Chauliognathus pensylvanicus* and *C. marginatus* [71]. Adult beetles feed on pollen and also form mating leks on flowers. Adult beetles infected with the conidial stage of the fungus become attached to flowers before death by grasping a flower tightly with their mandibles (Figure 5i). There are no fungal attachment structures such as rhizoids and the legs are not involved in grasping the plant. The fungus causes its hosts to grasp plants with their mandibles, but the mechanism behind this is unknown. Similar behavior is exhibited by “zombie ants” infected with *Ophiocordyceps* spp. (Ascomycota) [72]. It is thought that the fungus produes chemicals that manipulate the ant brain. Infected beetles producing conidia are held in the midst of feeding and mating adult beetles, where transmission of conidia to new hosts can occur. Research by Steinkraus et al. [71] indicated that primary conidia were not explosively discharged (but see Carner [73] for a system where *E. lampyridarum* conidia apparently were actively discharged).

By collecting large numbers of living adult *C. pensylvanicus* from flowers and holding them in the laboratory, it was shown that beetles producing resting spores stage fall to the ground, with no external growth of the fungus whatsoever (i.e., no cystidia, rhizoids, or conidiophores). Infected beetle abdomens were filled with dark brown resting spores (Figure 5j) and were friable and easily broken. Presumably the bodies break apart or are scavenged by other organisms and the resting spores are distributed on the soil under the flowering plants where the adults had fed and mated. There, they remain until the following spring or summer when they germinate and infect new soldier beetles.

#### 3.3.6. *Entomophaga maimaiga* and Gypsy Moth, *Lymantria dispar*

The locations of cadavers of gypsy moth, *Lymantria dispar*, larvae dying from *E. maimaiga* infections generally differ based on larval instar [74], which, in this host, also means that different types of spores are formed at different locations as conidia are produced from early instars and resting spores are usually produced from later instars [49]. Larvae of this species feed on leaves of many tree species and earlier instars are generally found in tree canopies. Conidia are actively ejected from cadavers of earlier instars killed by *E. maimaiga*, which are found with prolegs grasping the undersides of the wood of leaf-bearing twigs and the anterior portion of the body at a 90° angle to the twig, hanging vertically, with the head downward [75].

Later instar gypsy moth larvae have unusual behavior for lepidopteran larvae, as later instars climb trees every evening and descend in early morning to rest in darkened locations, often in the leaf litter, during the daylight hours; therefore, it is normal for later instars to walk up and down tree trunks each day [76]. Cadavers of later instar gypsy moth larvae that contain resting spores are usually found attached vertically to tree trunks, with prolegs extended horizontally and attached to the bark, and with heads downward (Figure 2) [75,77]. Thus, some aspects of the differential distribution of conidia versus resting spore producing cadavers are consistent with the normal locations for gypsy moth larvae of early versus later instars [74]. However, some pre-death behaviors, for example, early instar larvae grasping twigs and not being on leaves and later instars having prolegs extended laterally, could be caused by pre-death manipulation of larval behavior by the fungus.

## 4. Resting Spores in the Environment

### 4.1. Quantification of Resting Spores in Soil

A wet sieve method that may or may not be followed by density gradient separation has been routinely utilized for detection and quantification of resting spores of Entomophthoromycotina in the soil (e.g., [78,79,80,81,82]). Although this method is convenient to use, being adaptable to various species, it does not provide the specificity required when tracking an epizootic associated with a specific fungus of a given insect pest. The only way to track epizootics accurately and precisely is the application of molecular methods relying on species- or strain-specific primers. Thomsen and Jensen [83] designed species-specific primers that, in combination with a nested polymerase chain reaction (PCR) strategy, can be used to identify *E. muscae* resting spores. This two-step technique improved sensitivity and specificity of PCR assays when sampling DNA from in vivo samples (i.e., resting spores in host cadavers) [83]. In combination with real-time PCR, specific primers can also be used for quantitative detection of resting spores. So far, a species-specific real-time PCR assay has been developed for the resting spores of only one fungal species in the Entomophthoromycotina, *E. maimaiga* [84]. The primer pair and assay conditions developed by Castrillo et al. [84] were specific to *E. maimaiga* and were able to detect different strains of this species, but did not amplify the closely related *E. aulicae*. *Entomophaga maimaiga* is in the *E. aulicae* species complex [85]. A critical step in this method was breaking the thick walls of resting spores in soil samples to efficiently release fungal DNA. This was achieved through the use of high density beads such as silica and zirconia silica in combination with homogenization at high speeds (up to 5000 rpm) for 1 min. As the resting spores that were quantified were mixed in soil samples, the extracted fungal DNA samples were contaminated with DNA from other soil microorganisms and with soil organic matter. Humic acids present in the organic layer of the soil, where *E. maimaiga* resting spores are commonly found, can inhibit PCR reactions and affect assay sensitivity. Castrillo et al. [84] observed that at a high titer (10^4^ resting spores/g of soil), estimates from mixed DNA samples were close to actual titers in different soil types tested. At lower titers, however, estimates obtained were significantly lower in soil samples with high percentages of organic matter present.

### 4.2. Spatial Distribution and Densities in the Environment

The soil is generally considered the location where resting spores remain until receiving stimuli to germinate; therefore, resting spores must travel from infected hosts to the soil, but they have no independent means for transportation. Cadavers of cassava green mites, *Mononychellus tanajoa*, containing *Neozygites tanajoae* resting spores, are often attached to leaves by rhizoids. Cadavers then break apart while on top of leaves and resting spores are deposited on the leaf surface [43] (and see Figure 5d). Less commonly, *M. tanajoa* cadavers containing resting spores fall to the ground intact. In either case, resting spores are washed into the soil. Later instar gypsy moth larvae that die from *E. maimaiga* infections predominantly contain resting spores within cadavers. Many cadavers are initially found on tree trunks (see Section 3.3.6), where they dry and subsequently fall to the ground. Once cadavers start to decompose, resting spores are released from cadavers into the soil; this process usually requires 1–2 weeks. Low titers of resting spores also overwinter and survive on tree bark near the bases of trees [75].

The distribution of resting spores in the environment has been quantified for *E. maimaiga* infecting gypsy moth larvae. Resting spores are found in highest concentrations (mean = 4751 resting spores/g dry soil) in the organic layer of the soil, 0–10 cm from the bases of oak trees, with numbers decreasing precipitously with increasing distance from the tree trunk as well as with increasing depth in the soil [86]. Resting spores extracted from the bark of trees reached a maximum mean of 277 resting spores/25 cm^2^, much lower than than densities in the soil at the bases of trees. Gypsy moth larvae do not enter the soil and when larvae were exposed to soil with *E. maimaiga* resting spores buried 1 cm below the soil surface, they did not become infected (although exposure to this same sample of spores on the surface of the soil resulted in infection); therefore, the fact that resting spores predominantly remain at or near the soil surface is appropriate for this host.

Resting spores of *E. maimaiga* are also thought to be aggregated among trees; resting spore-bearing gypsy moth cadavers were found in larger numbers on tree species preferred by the host and not on non-preferred hosts. It is assumed that this distribution would result in greater densities of resting spores at the bases of preferred host trees [87].

### 4.3. Resting Spore Activity

#### 4.3.1. Dormancy and Germination

Resting spores are thought to normally be constitutively dormant after production and maturation [6,88], although *Conidiobolus thromboides* does not appear to go into dormancy [42]. It has been hypothesized that dormancy acts to synchronize resting spore germination with developing insect hosts [89]. Once resting spores are dormant, the thick wall resists stains and viability assessments have never been successful. Therefore, to date, the only way to determine whether or not resting spores are alive is to conduct trials to assess germination. However, the conditions necessary to break dormancy so that germination is possible have been determined for only eight species [42,45]. For seven of the eight species, periods of storage under colder temperatures varying from two weeks to nine months, and varying within and between species, are required before germination will begin. As an exception, *Zoophthora canadensis* requires a photoperiod of longer than 12 h of light per day for germination to begin. Exposure to hosts has not been shown to activate or accelerate germination of *E. maimaiga* resting spores [90]. There is a trend of more resting spores being responsive and germinating if the resting spores are older or have been stored at colder temperatures [89,91].

*Entomophaga maimaiga* resting spores produced under laboratory conditions and not allowed to dry after production did not go into dormancy and germinated asynchronously for at least 200 days [46]. When resting spores that had not dried were then stored at 4 °C for from 1 to 8 months, they also demonstrated this prolonged asynchronous germination.

To germinate, resting spores dissolve the large oil droplets and thick wall and one or two germ tubes are formed, which protrude through the epispore if one is present [45,89]. Germination does not require nutrients, generally has been demonstrated at temperatures of less than 20 °C, and is relatively slow and asynchronous. For *E. maimaiga* resting spores, more germination occurred with exposure to a photoperiod of 14:10 (light:dark) when compared with 13:11 or 12:12 [45]. 

#### 4.3.2. Horizontal Transmission Due to Resting Spores

Resting spores do not directly infect hosts. They germinate to produce infective germ conidia that can be actively ejected. In the case of *Entomophaga maimaiga*, the germ conidia look exactly like primary conidia produced externally from cadavers, although they are slightly smaller. However, for *Zoophthora radicans*, germinating resting spores produce capilliconidia that are not actively ejected. One resting spore has been shown to produce from one to five germ conidia and this varies by species. In *E. maimaiga*, when germ conidia infect, the fungus always produces only conidia in the resulting cadaver and not resting spores [42]. However, this was not found for *Furia gastropachae* as infections initiated when *Malacosoma disstria* larvae were exposed to soil containing resting spores usually produced either resting spores or conidia, with both types of spores produced in the same cadavers for <6% of infections [92].

Laboratory and field studies caging *L. dispar* larvae over soil with *E. maimaiga* have shown that resting spores begin germinating close to or slightly before *L. dispar* egg hatch (Table 3). *Lymantria dispar* overwinters as eggs and has one generation per year, with pupation in early July in northeastern North America. When larvae are caged over soil containing *E. maimaiga* resting spores throughout the larval season, studies conducted in numerous years have shown that levels of infection were correlated with moisture levels. When host populations were low, gypsy moth larvae were exposed to soil and *E. maimaiga* infections are assumed to have been initiated by resting spores. Infection levels from such soil exposures have been associated with temperatures between 15 and 25 °C [93], and have also been positively associated with resting spore density, gypsy moth density, canopy cover, and soil pH [94]. During some studies, infection levels among caged larvae ended in mid-June, but for one trial in 2010, infections continued into July (A.E.H. unpubl data). Studies with *F. gastropachae* demonstrated similar trends of greater infection when soil contained more moisture, although not when soil moisture was saturated [92].

#### 4.3.3. Persistence

Studies have demonstrated that many resting spores do not germinate the first year that they are produced, thus creating a pathogen reservoir in the environment for the next year. It has been hypothesized that *E. maimaiga* resting spores are dormant throughout the winter, after which the approximately two month period that larvae are present constitutes a windows for potential germination. However, if resting spores do not germinate for some reason during that time, for example, perhaps because of insufficient moisture or not being at the soil surface, they can re-enter dormancy until the next larval field season. The exact mechanism for long-term survival resulting in germination of only some resting spores during a period when hosts are present each year is not known. However, we know that resting spores persist in the environment. In small plots seeded with *E. maimaiga* resting spores, infection was documented six years after resting spore production [100]. Another study hypothesized that *E. maimaiga* resting spores created during epizootics 11–12 years prior to *L. dispar* soil exposures were responsible for infections [96]. However, studies have shown that over five years following epizootics, the titers of *E. maimaiga* resting spores in the soil generally decrease each year when gypsy moth populations are low [101], probably as a result of some resting spores germinating each year, but there is also the potential that mycoparasites are killing resting spores (see Section 4.4). For *F. gastropachae*, >500 resting spores/g dry soil were present four years after epizootics [92].

For *M. cicadina*, it has been thought that resting spores settled onto the ground and remained dormant for 13 or 17 years and were synchronized to germinate at the time of the mass emergence of cicada broods. However, Duke et al. [38] conducted an experimental study of *M. cicadina* resting spores from 17-year periodical cicadas captured in Iowa. The resting spores were sprayed on soil plots in an Arkansas forest that was due for an emergence of 13-year cicadas the next year. *Massospora cicadina* resting spores were capable of germinating and infecting *Magicicada tredecassini* after only one year of dormancy. This suggests that the resting spores of *M. cicadina* might be stimulated to germinate by proximity to mature periodical cicada nymphs and are not synchronized to germinate only after 13 or 17 years. We hypothesize that a chemical cue from periodical cicada nymphs could stimulate the resting spores to germinate and suggest that this should be tested experimentally.

### 4.4. Ecosystem Level Interactions

Resting spores allow long term persistence of fungi in the Entomophthoromycotina in the field [6], but their longevity or viability can be affected by abiotic (e.g., [46,99]) and biotic factors [102,103]). Interspecific interactions with other microorganisms in the soil can result in antagonism following contact. These microorganisms include fungi and fungi-like organisms (Oomycetes), collectively termed mycoparasites, that derive most or all of their nutrients from other fungi. Mycoparasitic relationships can be biotrophic (narrow host ranges, with complex, controlled, and relatively non-destructive interactions) or necrotrophic (broad host ranges, with unspecialized parasitic mechanism, and kill their host) [104]. The relationship, however, can change during the course of parasitism, from being biotrophic to necrotrophic, and can vary between hosts for a given mycoparasite. Mycoparasites have been described from Oomycetes (phylum Heterokontophyta) and different fungal phyla, more commonly in Ascomycota and Chytridiomycota for invasive necrotrophs [104]. Information on mycoparasites attacking entomopthoromycotan fungi is sparse, but is critical in understanding factors that affect the persistence and prevalence of resting spores in the soil and their horizontal transmission. A sampling of *E. maimaiga* from *L. dispar* cadavers by Hajek et al. [102] revealed azygospores parasitized by the chytrid *Gaertneriomyces semiglobifer*. Laboratory bioassays where *E. maimaiga* azygospores were exposed to this chytrid resulted in up to 82% parasitism 4 day post exposure and 94% after 8 day [102]. Although parasitism observed was higher in immature azygospores, the results showed that even thick-walled mature azygospores were susceptible to this mycoparasite. Resting spores are nutrient rich reservoirs and can be valuable resources for mycoparasitic fungi capable of penetrating their thick walls [105].

**Figure 6:**
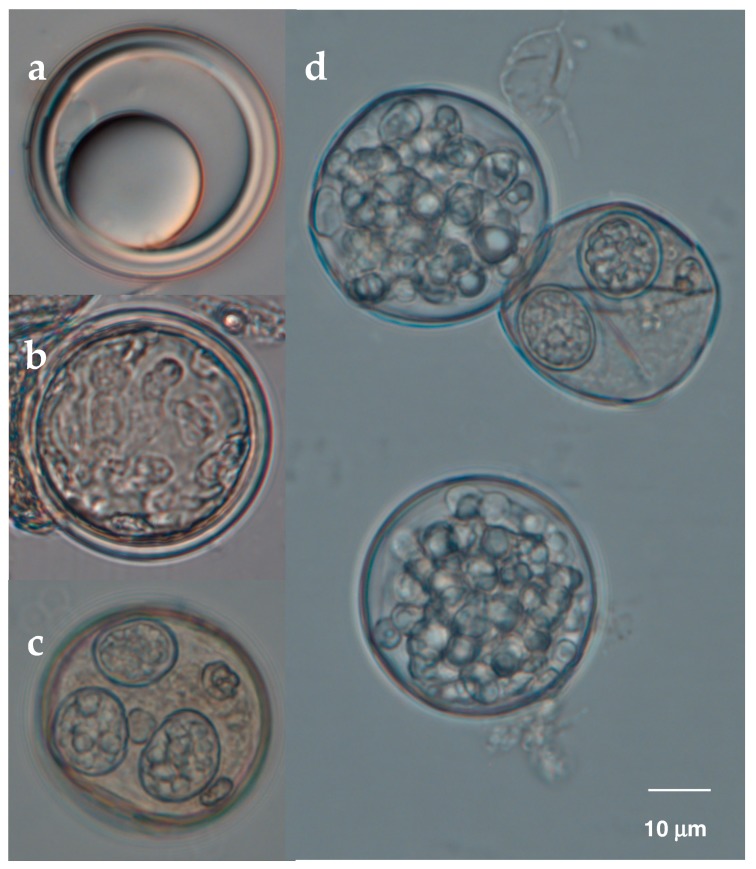
Healthy and mycoparasitized *Entomophaga maimaiga* resting spores. Parasitism was detected by comparing morphology of healthy spores (**a**) versus caged resting spores used as baits in soil samples from oak forests. Most of the resting spores used as bait were parasitized, misshapen, and filled with different fungal or fungi-like structures (**b**–**d**) indicating presence of multiple mycoparasitic species. (From [103]).

In a follow up survey on mycoparasites associated with *E. maimaiga* conducted by Castrillo et al. [103], the results revealed a high percentage (up to 90% or more) of parasitism in *E. maimaiga* resting spores in multiple sites tested. Using a combination of soil baiting and molecular methods, prevalence of mycopasitism was determined microscopically and presumptive mycoparasites were identified by PCR and sequencing of the ITS locus with taxon-specific primers. Evidence of parasitism included misshapen resting spores that were filled with distinct fungal structures of various forms (Figure 6). Microscopic examinations also suggested the possibility of multiple mycoparasites attacking a given resting spore. Identification of these presumptive mycoparasites revealed a *Pochonia* sp. (Ascomycota) and at least three *Pythium* species or strains (Oomycota). Given the various morphologically distinct fungal structures detected inside parasitized resting spores, it is likely that there were other mycoparasites not identified by the molecular method used. A few of the taxon-specific ITS primers tested had limited detection ranges [103]. Unlike the study by Hajek et al. [102], these presumptive mycoparasites were not cultured and details of their interaction with *E. maimaga* resting spores has not been studied in detail.

## 5. Use of Resting Spores for Control

Methods for in vitro production of resting spores have been developed for some entomophthoralean species (see table in [6]). Within *E. maimaiga*, there was extensive variability among different isolates regarding the numbers of resting spores that were formed in vitro [106]. Mass production methods were developed for a few aphid pathogenic species of *Conidiobolus* that readily make resting spores in vitro [6]. However, these methods did not result in marketable products.

As an alternative, resting spores of *E. maimaiga* have been obtained for control purposes by collecting gypsy moth cadavers filled with resting spores and releasing them in new areas. This method was used to hasten the spread of *E. maimaiga* in the eastern United States [107] and later in the northern Midwestern United States (A. Diss personal communication). Releasing resting spore-filled cadavers was also used to augment naturally occurring *E. maimaiga* in urban forests [108] and studies have demonstrated higher levels of infection by *E. maimaiga* when water was applied to resting spore-bearing soil at the bases of trees (e.g., [99]).

For classical biological control, cadavers containing resting spores have been successfully used to introduce *E. maimaiga* to Bulgaria in 1999 [109] and this method was then further used to spread *E. maimaiga* to more areas within Bulgaria (D. Pilarska pers. comm.).

## 6. Conclusions: Importance of Resting Spores to Epizootiology

Most species in the Entomophthoromycotina produce more than one kind of spore. The persistent resting spore stages are important to epizootiology, but have been under-studied in the majority of host/pathogen systems. This is caused in part by the fact that cadavers in which resting spores are produced are often in different locations than cadavers from which conidia are produced; thus, for many species within Entomophthoromycotina, the resting spores have not been described and these species are only known from the conidial stages. Also, the reverse occurs when resting spores have been found but associated conidial stages have not. Among species for which resting spores are known, there are relatively few instances where the biology and ecology have been investigated, although in the past few decades, this information has increased for specific systems. We hope that this review of our present knowledge about the biology and ecology of resting spores of fungi in the Entomophthoromycotina will encourage others to gain more knowledge about these essential stages about which we know so little. In particular, we encourage additional studies on resting spore dormancy, environmental conditions leading to resting spore germination, spread due to airborne infective germ conidia produced by resting spores, altered behaviors of insects before death when resting spores versus conidia are produced, and interactions with biological communities that impact environmental reservoirs of resting spores. In addition, the other life stages used by some species in this group for survival, such as hyphal bodies, deserve investigation, for similar reasons. 

## Figures and Tables

**Table 1:** Classification of subphylum Entomophthoromycotina (phylum Zoopagomycota) *.

Class	Order	Family	Genus (No. of Species) **	Examples (Habit/Hosts) [Reference]
Basidiobolomycetes	Basidiobolales	Basidiobolaceae	*Basidiobolus* (10)	*B. ranarum* (commensal in gut of frogs and toads) [22]
Neozygitomycetes	Neozygitales	Neozygitaceae	*Apterivorax* (2)	*A. acaricida* (mites) [23]
			*Neozygites* (20)	*N. fresenii* (aphids) [24] *N. floridana* (mites) [25]
			*Thaxterosporium* (1)	*T. turbinatum* (aphids) [26]
Entomophthoromycetes	Entomophthorales	Ancylistaceae	*Ancylistes* (5)	*A. closterii* (green algae) [27]
			*Conidiobolus* (55)	*C. macrosporus* (mosquitoes) [28] *C. obscurus* (aphids) [19]
			*Macrobiotophthora* (2)	*M. vermicola* (nematodes) [29]
		Completoriaceae	*Completoria* (1)	*C. complens* (plants) [30]
		Entomophthoraceae	*Batkoa* (10)	*B. apiculata* (lepidopterans, dipterans and homopterans) [16] *B. major* (coleopterans, dipterans, and homopterans) [16]
			*Entomophaga* (20)	*E. maimaiga* (gypsy moth) [31] *E. grylli* (grasshoppers) [32]
			*Entomophthora* (59)	*E. muscae* (flies) [33] *E. thripidum* (thrips) [34]
			*Erynia* (22)	*E. aquatica* (mosquitoes) [35] *E. conica* (blackflies) [36]
			*Furia* (17)	*F. gastropachae* (lepidopterans) [37]
			*Massospora* (14)	*M. cicadina* (cicadas) [38]
			*Orthomyces* (1)	*O. aleyrodis* (whiteflies) [39]
			*Pandora* (34)	*P. bullata* (flies) [40] *P. neoaphidis* (aphids) [41]
			*Strongwellsea* (3)	*S. castrans* (flies) [21]
			*Zoophthora* (41)	*Z. radicans* (lepidopterans and homopterans) [5]
		Meristacraceae	*Meristacrum* (2)	*M. milkoi* (flies) [30]

* Classification below the subphylum follows Humber’s proposal [8], but the subphylum and phylum are based on the proposal by Spatafora et al. [14]. ** Numbers of species based on Index Fungorum records (http://www.indexfungorum.org/Names/Names.asp), as of April 2018.

**Table 2:** Conditions associated with formation of resting spores in arthropod hosts.

Species	Host	Factors Associated with Resting Spore Formation (Type of Association)	Reference(s)
*Entomophaga maimaiga*	*Lymantria dispar*	Larval age (+), temperature (+), humidity (+), dose of inoculum (+), fungal isolate (variable)	[49,50]
*Pandora nouryi*	*Myzus persicae*	Temperature (−), dose of inoculum (+), photoperiod (+)	[51,52,53]
*Entomophthora muscae*	*Musca domestica*	Temperature(−), fungal isolate (variable)	[54]
*Entomophthora muscae*	*Delia radicum*	Photoperiod after midsummer (−), sex (+female)	[55]
*Zoophthora radicans*	*Therioaphis trifolii* f. *maculata*	Temperature (−), humidity (+), dose of inoculum (+), fungal isolate (variable)	[56]
*Neozygites floridana*	*Tetranychus urticae*	Temperature (−), photoperiod (−), light intensity (−)	[57]

**Table 3:** Bioassays conducted by exposing caged *Lymantria dispar* larvae to soil bearing resting spores of *Entomophaga maimaiga*.

Conditions	Results	Reference
Caged on soil (1 day exposures; 29 April–30 June, 24–26 July)	Infections occurred throughout April–June but not during July exposure; infections correlated with precipitation	[80]
Caged on soil (3–4 day exposures of different instars; 18 April–5 August 1994, 24 April–30 June 1995). Cages covered while in the field/local gypsy moth population very low.	First infections one week before egg hatch. Infections ending in mid-late June. Infection levels associated with soil moisture for different instars.	[45]
Caged on soil (2–5 day exposures; 27 April–25 June 1998)	Proportions infected associated with 5 day running totals of precipitation.	[95]
Caged on soil vs. on tree trunks vs. in the air within the forest canopy	Highest levels of infection occurred among larvae on the soil; this agreed with high levels of infection in the field and litter-dwelling behavior of later instars.	[96]
Caged on soil or in the forest canopy, comparing *L. dispar* infection with *Orgyia leucostigma* (2 day exposures beginning 17, 19 June, and 9 July)	Much more infection from soil than canopy exposures and more infection for *L. dispar* than *O. leucostigma*.	[97]
Caged on soil (2 day intervals; 4 April–8 May 1997 in Virginia); watered versus unwatered soil	Earliest infection was low (4–6 April), with egg hatch approx. 10 day later. Much more infection when soil beneath cages was watered.	[98]
Cages on soil (2001, 2002; tested from end May–beginning July). Resident gypsy moth populations very low.	Infection rates associated with precipitation in 2002 and temperature between 15 and 25 °C, atmospheric vapor pressure and precipitation in 2001.	[93]
Caged on soil and compared with lab (1999–2001; 4 day exposures). Resident gypsy moth populations low.	Infection rates associated with resting spore density, gypsy moth density, canopy cover, and soil pH (all soils were acidic). Cardinal direction not significant.	[94]
Caged on soil (2007–2009; early to late June in Pennsylvania)	Infection levels associated with moisture levels.	[99]
Caged on soil (1 June–19 July, 2010, in central New York where *L. dispar* populations were low).	Infections decreased after 25 June, but continued through 3 July, but not after.	Hajek et al. unpublished data
